# Environmental impact and phenotypic stability in potato clones resistant to late blight *Phytophthora infestans* (Mont) de Bary, resilient to climate change in Peru

**DOI:** 10.1371/journal.pone.0318255

**Published:** 2025-02-11

**Authors:** Manuel Gastelo, Carolina Bastos, Rodomiro Ortiz, Raúl Blas

**Affiliations:** 1 Postgraduate School, Universidad Nacional Agraria La Molina, Lima, Perú; 2 International Potato Center, Huancayo, Peru; 3 Swedish University of Agricultural Sciences department of Plant Breeding, Lomma, Sweden; 4 Department of Plant Science, Faculty of Agronomy, Universidad Nacional Agraria La Molina, Lima, Perú; ICAR - Indian Agricultural Research Institute, INDIA

## Abstract

Potato is one of the three most important foods in the world’s diet and is staple in the Peruvian highlands. This crop is affected by late blight, a disease that if not controlled in time can decimate production. The oomycete (*Phytophthora infestans*) causing this disease is controlled using fungicides, which affect the environment and human health, another form of control is the use of resistant cultivars. 30 potato clones from the LBHTC2 population were evaluated, with the objective of selecting clones with high levels of resistance to this disease, stable for tuber yield, low environmental impact and high economic profitability. The clones were planted in three field experiments in the 2021–2022 growing season. Two experiments with and without late blight chemical control in Oxapampa and Huánuco and one experiment under normal conditions of a potato crop in El Mantaro, Junin, using randomized complete blocks with three replications. The cultivars Yungay, Amarilis and Kory were used as controls for late blight resistance and tuber yield. Late blight resistance and environmental impact were determined based on experiments with and without control in Huánuco and Oxapampa. Yield stability and economic profitability were evaluated based on information from the three experiments. Clones CIP316375.102, CIP316361.187, CIP316367.117, CIP316356.149, CIP316367.147 were the ones that presented the highest yields, high Late blight resistance, phenotypically stable for tuber yield, with low environmental impact and high economic profitability, superior to control cultivars. These clones have high potential for sustainable production systems that allow reducing environmental impact, increasing economic profitability and improving producers’ living standards.

## Introduction

Potato (*Solanum tuberosum* L.) is the third most consumed food crop in the world after rice and wheat. In Peru, 330,000 ha are planted each year, contributing to farmers resilience and food security [1]. Per capita consumption in Peru has increased to 90 kg, which indicates that each year there is a greater source of income for small potato producers dedicated to family farming, a key part of sustainable rural development. Likewise, Peru has become the leading potato producer in Latin America and the Caribbean with 5.3 billion tons of potatoes per year, and a productivity of 16.5 t/ha [2], thus generating more than 110,000 jobs involving 711,313 families [3,4].

Late blight disease, caused by the oomycete *Phytophthora infestans* (Mont) de Bary, is the main threat to potato production worldwide [5,6]. When not controlled in a timely and adequate manner it can cause total loss of the crop. Due to climate change this disease can currently develop beyond 4000 meters of altitude, where it previously occurred sporadically [7]. Late blight is controlled by the frequent application of fungicides [8]. However, some strains show resistance to the fungicide metalaxyl [9]. Further, up to 20 fungicide applications are needed to control the disease in some potato production areas of Peru [10], causing a threat to the environment and human health. Another form of control is using cultivars with genetic resistance to late blight, which in addition to controlling the disease at economically profitable levels, reduce production costs, increase profitability, improving the quality of life of producers, and contribute to reducing the environmental impact and human health through reduced use of fungicides [11–14]. Integrated disease management is a control method that combines these two methods with cultural tasks such as planting density, fertilization, use of quality seed, and crop rotation.

Resistance to late blight can be vertical or specific to some races of the pathogen, and horizontal field resistance or not specific to any race. To select potato clones with horizontal resistance to late blight, it is necessary to conduct field trials in several environments to account for the variability of the pathogen and the existence of various isolates in endemic areas, such as Huánuco, Oxapampa that have adequate conditions to evaluate the incidence of late blight [15–18].

The use of fungicides in higher doses to control the late blight causes the pathogen to acquire resistance and spread rapidly in the crop plot and its neighbors, recommending replacing chemical control as the main control method due to the risk it represents for public health and its impact on the environment [19]. The environmental impact in the integrated control was found to be 2% of the conventional method in The Netherlands [20]. This difference was due to the choice of the agent, volume used and reduction of drift. In Ecuador, the susceptible cultivars Capiro and Superchola had the highest rates of environmental impact compared to the clones CIP387205.5 and CIP386209.10, whose resistant to late blight reduced the environmental impact rate by 92.22% [21]. The high environmental impact rate in these cultivars is attributed to the high use of fungicides to control the disease and that this can be reduced by using resistant cultivars that reduce the number of applications and the use of less toxic fungicides. The methodology to determine the Environmental Impact Rate (EIR) was developed by Cornell University and is an indicator to evaluate the potential risk of pesticide use in resistant clones compared to susceptible cultivars [22–24]. It is very important that genotypes with resistance to late blight are stable in tuber yield across various environments [25,26]. The yield and quality of tubers is influenced by genetic, environmental, agronomic management factors and the interrelationship between them. Several investigations show that the identification of superior genotypes is complicated by the genotype × environment (GE) interaction [27]. The analysis of GE interaction and the estimation of phenotypic stability has been studied in many crops [28]. There are many statistical methods to study the GE interaction and phenotypic stability in potato [29,30]. The multivariate method additive effects and multiplicative interaction (AMMI) combines the analysis of variance with the GE interaction effects and presents the results in biplots that allow a better identification of the most phenotypically stable genotypes [31–33]. Research carried out in Cuba and Uganda using the AMMI model demonstrated that this method is very useful in potato cultivation to identify the most stable genotypes [34,35].

The objective of this study was to determine the environmental impact and phenotypic stability in potato clones with resistance to late blight and to select clones with high levels of resistance to late blight, low environmental impact, phenotypically stable for tuber yield and high economic profitability.

## Materials and methods

Thirty potato breeding clones belonging to the LBHTC2, developed by the genetic improvement program of the International Potato Center (CIP) were evaluated. These elite clones were selected for their resistance to late blight from 2017 to 2021, under natural disease conditions in Oxapampa at 1850 masl, where the environmental conditions of temperature (15–19^o^C), relative humidity >  80% and precipitation ( > 1000 mm per year), are optimal to induce a high disease pressure, in addition to the presence of complex isolates of *P. infestans*, Resistance was determined based on low AUDPC and sAUDPC values. Three cultivars were used as controls, namely Amarilis (moderately resistant), Yungay (susceptible) and Kory (resistant) (Table 1).

**Table 1:** Clones of the LBHT population cycle 2 with resistance to Late blight used in this study 2021–2022.

#	Clone	Female parent	Male parent	#	Clone	Female parent	Male parent
1	CIP316344.165	CIP398098.570	CIP398208.219	18	CIP316361.191	CIP398201.510	CIP398208.620
2	CIP316346.204	CIP398192.553	CIP398208.219	19	CIP316361.209	CIP398201.510	CIP398208.620
3	CIP316352.122	CIP398098.203	CIP398208.219	20	CIP316361.244	CIP398201.510	CIP398208.620
4	CIP316352.152	CIP398098.203	CIP398208.219	21	CIP316365.166	CIP304081.44	CIP398208.620
5	CIP316353.148	CIP398190.200	CIP398208.219	22	CIP316367.117	CIP398190.200	CIP398208.620
6	CIP316353.741	CIP398190.200	CIP398208.219	23	CIP316367.118	CIP398190.200	CIP398208.620
7	CIP316354.112	CIP398208.505	CIP398208.219	24	CIP316367.134	CIP398190.200	CIP398208.620
8	CIP316354.169	CIP398208.505	CIP398208.219	25	CIP316367.147	CIP398190.200	CIP398208.620
9	CIP316355.162	CIP398208.670	CIP398208.219	26	CIP316367.148	CIP398190.200	CIP398208.620
10	CIP316356.149	CIP302551.26	CIP398208.219	27	CIP316367.177	CIP398190.200	CIP398208.620
11	CIP316358.214	CIP398098.65	CIP398208.620	28	CIP316375.101	CIP398201.510	CIP398203.5
12	CIP316360.241	CIP398192.553	CIP398208.620	29	CIP316375.102	CIP398201.510	CIP398203.5
13	CIP316361.118	CIP398201.510	CIP398208.620	30	CIP316387.156	CIP398192.553	CIP398208.33
14	CIP316361.121	CIP398201.510	CIP398208.620	31	Amarilis		
15	CIP316361.158	CIP398201.510	CIP398208.620	32	Kory		
16	CIP316361.187	CIP398201.510	CIP398208.620	33	Yungay		
17	CIP316361.190	CIP398201.510	CIP398208.620				

The trials were planted during the 2021–2022 growing season, under natural field conditions. In Oxapampa and Huánuco two trials were planted, one with late blight control (Experiment 1) and another without control (Experiment 2), which are two sites with optimal environmental conditions (precipitation, temperature and relative humidity) for a high disease pressure, in addition to a great variability of the pathogen [16,36]. A third trial was carried out in El Mantaro (Junín) under normal conditions of a marketable potato production field (Experiment 3), (Table 2). The two experiments with and without late blight control were planted in Huánuco on 22^nd^ September 2021, and harvested on 10^th^ February 2022, and in Oxapampa the planting was on 1^st^ October 2021, and the harvest was on 1^st^ February 2022. The randomized complete block design was used with three replications of 10 plants per clone. The synthetic fertilizer dose of 180–200–160 kg of NPK per hectare was used, being urea (46% N) the source of Nitrogen, diammonium phosphate (46% P_2_O_5_,18% N) the phosphorus source, and potassium sulfate (50% K_2_O) the source of potassium. Pest control was that of a marketable potato crop.

**Table 2:** Sites where the trials were carried out. 2021–2022 growing season.

Site	Experiment	Altitude masl	Latitude	Longitude	Temperature average ºC	Relative humidity %	Rainfall mm
Oxapampa	1,2	1850	10°34′48″ S	75°24′0″ W	18.54	88.74	660
Huanuco	1,2	2110	9°48′5.9″ S	76°4′13.26″ O	14.76	84.42	433
El Mantaro	3	3320	11°49′20″S	75°23′31″O*	11.00	71.00	316

* Temperature, relative humidity and precipitation of the meteorological station installed during the period of field tests Source: CLIMATE-DATA.ORG (http://es.climate-data.org/location/4353/).

MINEM (http://www.minem.gob.pe/minem/archivos/file/DGGAE/ARCHIVOS/estudios/EIAS%20-%20hidrocarburos/108/EIA%20DIGITAL/Cap%203A%20LB%20Fisicoquimico/Cap%203A%20Texto.p).

In experiment 1, a contact fungicide (Mancozeb) was applied twice up to 35 days after planting to the clones and control varieties. In experiment 2 with late blight control, contact and systemic fungicides (Mancozeb, Cymoxanil and Propineb) were applied to the clones and varieties in an appropriate and timely manner according to the present environmental conditions and their resistance. The experiment 3 carried out in El Mantaro was planted on 27^th^ November 2021 and harvested on 20^th^ April 2022, No fungicide applications were carried out. In all experiments at harvest, the number of plants, as well as the number and weight of marketable and non-marketable tubers in kg per experimental unit were recorded. With these values, the marketable and total yield per hectare.

### Selection of clones resistant to late blight

30 elite clones previously evaluated for late blight resistance from 2017–2021 were evaluated. Late blight resistance was determined and validated based on the evaluations of the damage caused by late blight in the uncontrolled experiments, planted in Huánuco and Oxapampa, the AUDPC [37] and sAUDPC were calculated as parameters of the resistance of the clones. The additive linear model used for the analysis of variance was the following:


$${\text{Y}}_{\text{ij}}=\text{µ}+{\text{α}}_{\text{i}}+{\text{k}}_{\text{j}}+{\text{ε}}_{\text{ij}}$$

where Y_ij_ is the value in the plot corresponding to the i^th^ genotype in the j^th^ block, μ is the general mean, α_i_ is the effect of the i^th^ genotype, k_j_ is the effect of j^th^ block, and ε_ij_ is the experimental error (pure and residual) associated with observation Y_ij_.

The AUDPC was estimated as:


$$\text{AUDPC}=\sum_{\text{i}=1}^{\text{n}}\frac{{\text{X}}_{\text{i}+1}+{\text{X}}_{\text{i}}}{2}\times \left({\text{T}}_{\text{i}+1}-{\text{T}}_{\text{i}}\right)$$

where Xi is the percentage of infection at i days after planting, Xi + 1 is the percentage of infection at i +  1 days after planting, and (Ti + 1 − Ti) is the number of days between late blight evaluations and n is the number of evaluations.

The late blight susceptibility scale (sAUDPC) has values from 0 to 9, with 0 being a very resistant genotype and 9 being very susceptible [38], and is calculated using the following equation:


$$\text{Sx}=\text{Sy }\left(\text{Dx}/\text{Dy}\right)$$

where Sx is the scale value calculated for the clone under study; Sy is the scale value (6) assigned to the susceptible control cultivar Yungay, Dx is the AUDPC value of the clone under study, and Dy is the AUDPC value of the susceptible control.

The analysis of variance and Tukey’s mean comparison test at 5% (*P* =  0.05) were performed for AUDPC, sAUDPC, marketable yield and total tubers per hectare. The statistical software SPSS version 25, R version 4.3.2 and Microsoft Excel 97-2003 were used for statistical analysis.

### Environmental impact rate (EIR)

The information obtained in experiment 2 planted in Huánuco and Oxapampa was used to determine the environmental impact rate (EIR). The name of the fungicide used was recorded with its environmental impact coefficient (EIC), number of applications (NA), dose (D) and percentage of active ingredient (PAI) [22,24]. Environmental impact rate (EIR) was determined using the following formula [22]:


EIR = EIC  × PAI  × D × NA

The environmental impact coefficient (EIC) was determined using the following formula:


$$\text{EIC }=\left\{\text{C }\left[\left(\text{DT }5\right)+\left(\text{DT P}\right)]+[\left(\text{C }\left(\left(\text{S }+\text{P}\right)/2\right)\text{SY}\right)+\left(\text{L}\right)]+[\left(\text{F R}\right)+\left(\text{D }\left(\left(\text{S }+\text{P}\right)/2\right)3\right)+\left(\text{Z P }3\right)+\left(\text{B P }5\right)\right]\right\}/3$$

where C is chronic toxicity, DT is dermal toxicity, P is half-life on plant surface, S is residue half-life in soil, SY is systematicity, L is leaching potential, F is fish toxicity, R is surface loss potential, D is bird toxicity, Z is bee toxicity, and B is toxicity to beneficial arthropods.

### Economic profitability

Economic Profitability was determined based on yield, production costs and farm sale price for each location and then calculated from the average value and sensitivity analysis of costs and yields using the following formulas:


Total income=Tuber yield per hectare  x farm sale price.

Net income =Total income –production costs

$$\text{Economic profitability}\left(\text{\%}\right)=\left(\text{Net income}/\text{production costs}\right)100$$

The farm sale price is S/1.00 for the Yungay, Amarilis and breeding clones, for the Kory, el precio de venta fue S/ 0.70 which has a lower price due to its high glycoalkaloid content induced low quality.

The selection of the resistant elite clones was based on their AUDPC and sAUDPC, which should have been at least lower than the resistant Kory cultivar control, the marketable and total yield per hectare on average in the two locations, higher than Kory, with a low environmental impact rate and high economic profitability.

### Phenotypic stability of the marketable yield of tubers

The phenotypic stability of 30 potato clones and three control cultivars planted In experiments 2 and 3, was determined using the AMMI model [39,40], which integrates the analysis of variance, analysis of the principal components (PCs), the SVAMMI stability value AMMI [41]and the marketable yield stability index (MYSI) [42]. The model used for the analysis of variance was the following:


$${\text{Y}}_{\text{ij}}=\text{µ}+{\text{g}}_{\text{i}}+{\text{e}}_{\text{j}}+\sum {\text{λ}}_{\text{k}}{\text{α}}_{\text{ik}}{\text{γ}}_{\text{jk}}+\epsilon_{\text{ij}}$$

where, Y_ij_ is the marketable performance of the i^th^ clone in the j^th^ environment, g_i_ is the effect of the i^th^ clone, e_j_ is the effect of the j^th^ environment, λ_k_ =  is the square root of the value of the k^th^ principal component, α_ik_ and γ_jk_ are the k^th^ principal component of the i^th^ clone in the j^th^ environment, respectively, and ε_ij_ =  is the experimental error (pure and residual).

The SVAMMI value was used as a quantitative measure of clone stability for marketable tuber yield using the formula proposed by [41]; a clone is considered stable when its SVAMMI value is low.


$$\text{SVAMMI }=\left(\left(\text{sum of squares PC}1/\text{sum of squares PC}2\right)\left(\text{value PC}1\right)2+\left(\text{value PC}2\right)2\right)\text{½}$$

To select a stable clone with high marketable tuber yield, the Marketable Yield Stability Index (MYSI) was used. The lower this value indicates that the clone is stable with high marketable tuber yield. The **MYSI** was calculated as follows [42]:


MYSI =RSVAMMI +RMY

where RSVAMMI is the Ranking of SVAMMI and RMY is the ranking of marketable yield. A clone was considered stable when its MYSI was lower than that of the Yungay cultivar.

## Results

### Late blight resistant breeding clones

In the experiment 1, there were statistically significant differences (α = 0.01) between the clones for AUDPC, sAUDPC, marketable yield and total yield, thereby indicating that the clones presented different levels of resistance and tuber yield both in Huánuco and in Oxapampa. The coefficients of variability were low (Table 3).

**Table 3:** Analysis of variance by location for area under disease progress curve (AUDPC), late blight susceptibility scale (sAUDPC), marketable and total tuber yield (t/ha), without and with late blight control (Huánuco and Oxapampa, 2021–2022).

Source of variation	DF^1^	Mean Square Without Control							
		Huánuco				Oxapampa			
		AUDPC	sAUDPC	Marketable tuber yield	Total tuber yield	AUDPC	sAUDPC	Marketable tuber yield	Total tuber yield
Repetitions	2	1588.13	0.02	52.78	67.61	4457.10	0.06	146.09^**^	150.56^**^
Clones	32	254114.84^**^	3.195^**^	643.52 ^**^	816.45^**^	292319.97^**^	3.68^**^	545.62^**^	569.63^**^
Error	64	1209.23	0.02	59.91	47.74	2201.96	0.03	23.55	25.78
C.V. %		20.65	20.37	17.75	13.47	11.10	11.15	13.56	13.03
Source of variation	DF^1^	Mean Square With control							
		Huánuco				Oxapampa			
		AUDPC	sAUDPC	Marketable tuber yield	Total tuber yield	AUDPC	sAUDPC	Marketable tuber yield	Total tuber yield
Repetitions	2	0.01	0.12	402.92^**^	804.88^**^	6093.91^**^	30.08^**^	11.44	27.99
Clones	32	186.90^**^	4.17^**^	391.19^*^	482.45^*^	821.1^**^	40.61^**^	254.6^**^	233.32^**^
Error	64	15.96	0.08	76.24	92.38	804.98	3.46	34.59	39.9
C.V. (%)		28.6	25.7	17.43	15.99	18.9	17.58	15.66	4.69

^1^ DF: degrees of freedom.

** indicates significant source of variation for clones at *P* ≤  0.01.

* indicates significant source of variation for clones at *P* ≤  0.05.

**Fig 1:**
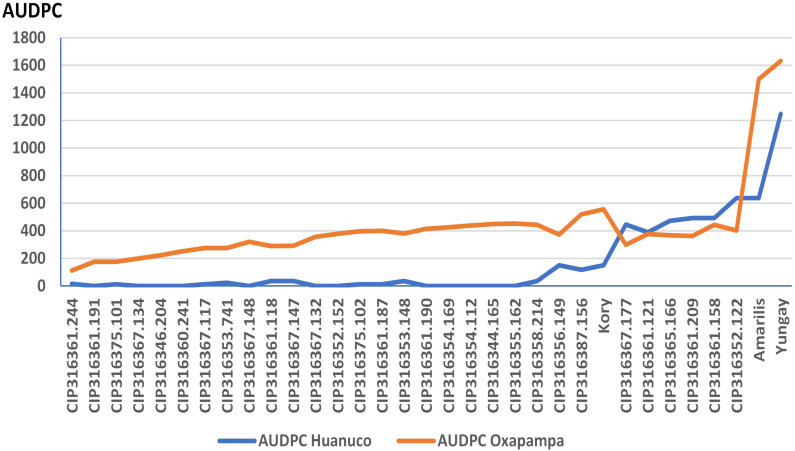
Resistance to late blight (AUDPC) in potato clones. Huánuco and Oxapampa 2021–2022.

In Huanuco twenty-three of the 30 breeding clones had lower AUDPC and sAUDPC values than the resistant Kory with 150 and 0.53 of AUDPC and sAUDPC respectively. In Oxapampa, the AUDPC of the clones ranged from 111 to 519, all with values lower than the resistant Kory (AUDPC =  556). The sAUDPC values of the clones ranged from 0.39 to 1.84, which were lower than that of Kory (1.97). The susceptible cultivar Yungay had the highest AUDPC in Huanuco and Oxapampa with 1248 and 1633 respectively, in both sites, the susceptible Yungay had a sAUDPC of 6 (Table 4, Fig 1).

**Table 4:** Tukey mean comparison test (α = 0.05) for area under disease progress curve (AUDPC), late blight susceptibility scale (sAUDPC), without late blight control (Huánuco and Oxapampa 2021–2022).

Clone	Huánuco				Oxapampa			
	AUDPC	α = 0.05	sAUDPC	α = 0.05	AUDPC	α = 0.05	sAUDPC	α = 0.05
CIP316344.165	0	a	0.00	a	450	hij	1.59	hij
CIP316346.204	0	a	0.00	a	222	bc	0.79	abc
CIP316352.122	637	e	2.26	e	403	defghij	1.43	defghij
CIP316352.152	0	a	0.00	a	378	defghi	1.34	defghi
CIP316353.148	35	ab	0.12	ab	379	defghi	1.35	defghi
CIP316353.741	23	ab	0.08	ab	274	bcde	0.97	bcde
CIP316354.112	0	a	0.00	a	438	fghij	1.55	fghij
CIP316354.169	0	a	0.00	a	426	efghij	1.51	efghij
CIP316355.162	0	a	0.00	a	452	hij	1.60	hij
CIP316356.149	150	c	0.53	c	374	cdefghi	1.33	cdefghi
CIP316358.214	35	ab	0.12	ab	444	ghij	1.57	ghij
CIP316360.241	0	a	0.00	a	251	bcd	0.89	abcd
CIP316361.118	35	ab	0.12	ab	289	bcdef	1.02	bcdef
CIP316361.121	390	d	1.38	d	376	defghi	1.33	defghi
CIP316361.158	492	d	1.74	d	443	fghij	1.57	fghij
CIP316361.187	12	ab	0.04	ab	399	defghi	1.42	defghi
CIP316361.190	0	a	0.00	a	415	efghij	1.47	efghij
CIP316361.191	0	a	0.00	a	175	ab	0.62	ab
CIP316361.209	493	d	1.75	d	362	cdefgh	1.28	cdefgh
CIP316361.244	17	ab	0.06	ab	111	a	0.39	a
CIP316365.166	472	d	1.67	d	368	cdefghi	1.30	cdefghi
CIP316367.117	12	ab	0.04	ab	274	bcde	0.97	bcde
CIP316367.118	0	a	0.00	a	356	cdefgh	0.70	ab
CIP316367.134	0	a	0.00	a	199	ab	1.03	bcdefg
CIP316367.147	35	ab	0.12	ab	291	bcdefg	1.14	bcdefgh
CIP316367.148	0	a	0.00	a	321	bcdefgh	1.26	cdefgh
CIP316367.177	445	d	1.58	d	298	bcdefgh	1.06	bcdefgh
CIP316375.101	12	ab	0.04	ab	175	ab	0.62	ab
CIP316375.102	12	ab	0.04	ab	397	defghi	1.41	defghi
CIP316387.156	117	bc	0.41	bc	519	ij	1.84	ij
Kory	150	c	0.53	c	556	j	1.97	j
Amarilis	752	f	2.67	f	1499	k	5.32	k
Yungay	1248	g	6.00	g	1633	k	6.00	k
Standard deviation	20.08		0.08		27.09		0.10	

Different letter after the mean value indicates a significant difference according to Tukey test (P < 0.05).

The marketable tuber yield in Huánuco ranged from 18.70 to 65.86 t/ha. Twenty-three clones had higher yield than the resistant Kory (37.65 t/ha). The susceptible Yungay had a yield of 9.01 t/ha. In Oxapampa, the clones had a marketable tuber yield ranging from 13.98 to 59.86 t/ha. Twenty-nine of them were superior in yield than Kory (17.05 t/ha). The susceptible Yungay yielded 1.02 t/ha (Table 4). On average, the marketable yield of the breeding clones was 45.68 t/ha Huánuco, higher than the yield in Oxapampa (38.47 t/ha), which could be because Huánuco had less late blight infection than Oxapampa, as indicated by the AUDPC values (Table 6). Resistant clones tend to become more infected as environmental conditions become favorable for late blight, but without reaching AUDPC values that affect yields economically. Susceptible clones on the other hand, when exposed to high disease pressure, can reach 100% infection and therefore total yield loss.

In the experiment 2, there were statistically significant differences (α = 0.01) between the clones for AUDPC, sAUDPC in both sites Huanuco and Oxapampa, statistically significant differences were found in Huánuco (α = 0.05) and in Oxapampa (α = 0.01) for marketable and total tuber yield (Table 3).

Marketable tuber yield in Huánuco ranged from 25.12 to 67.04 t/ha. Twenty breeding clones had higher yields than Kory, Amarilis and Yungay. In Oxapampa, the marketable yield ranged from 20.06 to 57.41 t/ha. Twenty-six clones had higher yields than the control cultivars, whose yields were high because late blight was controlled in a timely and adequate manner. The increase in yields in most clones was not significant when the disease was controlled. On average, in Huánuco and Oxapampa, marketable yields increased by 16.29 and 1.72% respectively, thereby showing the effect of their different levels of resistance. In the susceptible Yungay the increase was significant in Huánuco and Oxapampa; i.e., 404.11% (or 9.01 to 45.43 t/ha), and 2701.18 (or 1.02 to 28.70 t/ha), respectively. In the moderately resistant Amarilis, the increase was also significant but in a lower percentage than Yungay. In Huánuco, the marketable yield increased by 242.79% (or from 14.14 to 48.46 t/ha) and in Oxapampa it was 371.60% (or 6.09 to 28.70 t/ha). There is a high correlation between AUDPC values and marketable tuber yield increases (r =  0.75, Pearson correlation p <= 0.01) (Table 5),

**Table 5:** Marketable and total tuber yield, without and with late blight control in Huánuco and Oxapampa (2021–2022).

Clone	Marketable tuber yield t/ha										Total tuber yield t/ha									
	Huanuco				Increase %	Oxapampa				Increase %	Huanuco				Increase %	Oxapampa				Increase %
	Without control	α = 0.05	With control	α = 0.05		Without control	α = 0.05	With control	α = 0.05		Without control	α = 0.05	With control	α = 0.05		Without control	α = 0.05	With control	α = 0.05	
CIP316367.117	57.35	abcde	58.09	abcd	1.29	59.86	a	57.41	a	−4.10	62.90	abcdefg	63.21	abcd	0.49	62.21	a	61.11	a	−1.77
CIP316361.187	61.30	abc	62.96	a	2.72	52.64	abc	52.90	ab	0.49	70.12	abcd	64.69	abcd	−7.75	57.09	ab	56.85	ab	−0.41
CIP316360.241	33.64	efghij	42.16	abcde	25.32	56.72	ab	50.37	abcd	−11.19	38.89	ghijk	52.41	abcd	34.76	60.85	a	54.20	abc	−10.94
CIP316346.204	54.07	abcdef	55.06	abcde	1.83	50.44	abcd	51.73	abc	2.55	56.67	abcdefghi	61.23	abcd	8.06	53.47	abc	53.21	abcd	−0.48
CIP316375.102	65.86	a	67.04	a	1.78	47.44	abcde	48.27	abcde	1.74	78.09	ab	84.88	a	8.70	50.47	abcd	52.10	abcde	3.23
CIP316365.166	31.11	fghij	32.53	bcde	4.56	49.77	abcd	47.04	abcdef	−5.48	35.74	hijk	39.32	cd	10.02	51.86	abcd	51.36	abcde	−0.98
CIP316367.134	63.58	ab	64.01	a	0.68	46.63	abcdef	39.88	abcdefgh	−14.48	78.46	a	77.04	ab	−1.81	55.15	abc	50.74	abcde	−7.99
CIP316361.158	32.96	efghij	42.22	abcde	28.09	44.17	abcdefg	45.99	abcdef	4.11	36.42	hijk	47.16	bcd	29.49	46.70	abcdef	49.94	abcde	6.93
CIP316361.191	54.75	abcdef	58.70	abc	7.22	43.51	abcdefgh	44.94	abcdefg	3.29	61.79	abcdefg	73.46	abc	18.88	47.77	abcde	49.01	abcdef	2.61
CIP316356.149	52.47	abcdef	57.10	abcd	8.82	37.17	cdefghij	44.57	abcdefg	19.89	56.98	abcdefghi	62.72	abcd	10.08	39.15	cdefgh	47.72	abcdefg	21.89
CIP316361.244	59.44	abcd	32.78	bcde	−44.86	48.58	abcde	44.32	abcdefg	−8.77	65.06	abcdefg	45.62	bcd	−29.89	52.04	abc	46.98	abcdefg	−9.73
CIP316367.147	45.52	abcdefg	66.67	a	46.46	42.36	bcdefghi	39.94	abcdefgh	−5.71	73.89	abc	74.81	ab	1.25	47.36	abcde	43.70	abcdefgh	−7.72
CIP316355.162	40.80	bcdefgh	57.84	abcd	41.75	38.06	cdefghij	39.38	abcdefgh	3.47	48.15	cdefghi	61.67	abcd	28.08	40.22	bcdefgh	43.33	abcdefgh	7.73
Yungay	9.01	j	45.43	abcde	404.11	1.02	n	28.70	fghi	2701.18	14.88	k	58.70	abcd	294.58	4.17	k	43.15	abcdefgh	934.03
CIP316361.190	46.05	abcdefg	47.65	abcde	3.49	37.11	cdefghij	38.52	bcdefghi	3.79	67.72	abcdef	75.56	ab	11.58	41.49	bcdefgh	42.35	abcdefgh	2.05
CIP316352.122	18.70	hij	50.43	abcde	169.64	32.33	efghijk	37.16	bcdefghi	14.93	23.58	jk	52.90	abcd	124.35	34.37	defghi	41.73	abcdefgh	21.41
CIP316361.118	52.78	abcdef	55.06	abcde	4.33	37.54	cdefghij	38.64	abcdefghi	2.93	57.53	abcdefghi	70.06	abc	21.78	38.59	cdefghi	41.60	abcdefgh	7.81
CIP316367.118	60.99	abc	61.73	ab	1.21	37.17	cdefghij	35.12	bcdefghi	−5.51	68.95	abcde	75.80	ab	9.94	40.51	bcdefgh	41.05	abcdefgh	1.34
CIP316367.177	43.27	abcdefgh	60.74	ab	40.37	41.67	bcdefghi	36.91	bcdefghi	−11.41	47.41	defghi	70.43	abc	48.57	45.49	abcdefg	39.81	bcdefgh	−12.48
CIP316353.741	47.59	abcdefg	43.09	abcde	−9.47	37.46	cdefghij	35.93	bcdefghi	−4.09	60.86	abcdefgh	59.69	abcd	−1.93	40.17	bcdefgh	39.57	bcdefgh	−1.51
CIP316367.148	54.69	abcdef	61.48	ab	12.42	35.40	defghij	34.88	bcdefghi	−1.47	59.20	abcdefgh	69.51	abc	17.41	40.02	bcdefgh	38.83	bcdefgh	−2.99
CIP316354.112	38.21	cdefghi	56.85	abcd	48.79	26.48	ijkl	31.85	defghi	20.28	45.74	efghij	65.00	abcd	42.11	28.89	ghij	38.52	bcdefgh	33.33
CIP316361.121	24.63	ghij	28.46	de	15.54	35.95	cdefghij	36.73	bcdefghi	2.16	29.44	ijk	33.89	d	15.09	38.05	cdefghi	38.40	bcdefgh	0.91
CIP316361.209	20.06	hij	29.20	cde	45.54	35.51	defghij	33.33	cdefghi	−6.12	25.62	ijk	38.89	cd	51.81	37.91	cdefghi	38.33	bcdefgh	1.11
CIP316353.148	52.65	abcdef	53.77	abcde	2.11	35.78	cdefghij	36.23	bcdefghi	1.28	59.14	abcdefgh	61.73	abcd	4.38	37.69	cdefghi	37.72	bcdefgh	0.07
CIP316344.165	34.57	defghi	25.12	e	−27.32	26.72	hijkl	28.83	fghi	7.90	43.40	fghijk	32.90	d	−24.18	31.47	efghij	35.19	cdefgh	11.81
Amarilis	14.14	ij	48.46	abcde	242.79	6.09	mn	28.70	fghi	371.60	23.77	jk	55.49	abcd	133.51	10.72	jk	34.44	cdefgh	221.43
CIP316354.169	49.81	abcdef	53.95	abcde	8.30	28.20	ghijkl	29.69	efghi	5.30	54.38	bcdefghi	60.06	abcd	10.44	29.68	fghij	33.52	defgh	12.94
Kory	37.65	cdefghi	41.85	abcde	11.15	17.05	klm	25.43	hi	49.17	46.73	defghi	64.94	abcd	38.97	21.21	hijk	32.22	efgh	51.92
CIP316358.214	50.00	abcdef	52.72	abcde	5.43	26.72	hijkl	27.10	ghi	1.43	54.57	bcdefghi	57.78	abcd	5.88	29.25	fghij	29.51	fgh	0.89
CIP316352.152	37.84	cdefghi	40.86	abcde	7.99	21.93	jklm	22.65	hi	3.32	43.70	fghijk	52.28	abcd	19.63	24.27	hij	29.44	fgh	21.31
CIP316375.101	48.09	abcdefg	50.56	abcde	5.13	29.79	fghijk	26.11	ghi	−12.35	62.53	abcdefg	66.36	abcd	6.12	32.32	efghij	28.52	gh	−11.76
CIP316387.156	37.72	cdefghi	48.89	abcde	29.62	13.98	lm	20.06	i	43.55	40.93	fghijk	52.65	abcd	28.66	14.96	ijk	23.52	h	57.18
Average clones	43.59		50.44		36.80	37.18		39.02		104.64	51.53		60.38		30.49	40.47		43.54		42.88
Average varieties	41.21		46.77		14.25	21.90		22.94		11.51	49.05		57.10		18.14	23.85		27.16		22.24
Standard deviation	4.47		5.04			2.80		3.40			3.99		5.55			2.93		3.65		

Different letter after the mean value indicates a significant difference according to Tukey test (P < 0.05).

### Environmental impact rate (EIR)

Late blight control in clones was carried out with Mancozeb, a contact fungicide, applied 2 to 4 times according to the presence of disease symptoms in the clones. In control varieties, late blight was controlled with applications of Mancozeb 2 to 5 times and a systemic fungicide based on cymoxanil and propineb 6 to 7 times during the vegetative period (Table 6).

**Table 6:** Environmental Impact Rate in clones and varieties control in Huánuco and Oxapampa 2021–2022.

Clone	Number of Applications		Environmental Impact Rate (EIR)									Reduction of EIR (%)
			Huánuco				Oxapampa				Average	
	Huánuco	Oxapampa	Mancozeb	Cymoxanil	Propineb	Total	Mancozeb	Cymoxanil	Propineb	Total		
CIP316367.147	Mancozeb = 2	Mancozeb = 2	46.72	0.00	0.00	46.72	46.72	0.00	0.00	46.72	46.72	0.82
CIP316352.152	Mancozeb = 2	Mancozeb = 3	46.72	0.00	0.00	46.72	70.08	0.00	0.00	70.08	58.40	0.77
CIP316353.741	Mancozeb = 2	Mancozeb = 3	46.72	0.00	0.00	46.72	70.08	0.00	0.00	70.08	58.40	0.77
CIP316354.169	Mancozeb = 2	Mancozeb = 3	46.72	0.00	0.00	46.72	70.08	0.00	0.00	70.08	58.40	0.77
CIP316356.149	Mancozeb = 2	Mancozeb = 3	46.72	0.00	0.00	46.72	70.08	0.00	0.00	70.08	58.40	0.77
CIP316358.214	Mancozeb = 2	Mancozeb = 3	46.72	0.00	0.00	46.72	70.08	0.00	0.00	70.08	58.40	0.77
CIP316360.241	Mancozeb = 2	Mancozeb = 3	46.72	0.00	0.00	46.72	70.08	0.00	0.00	70.08	58.40	0.77
CIP316361.118	Mancozeb = 2	Mancozeb = 3	46.72	0.00	0.00	46.72	70.08	0.00	0.00	70.08	58.40	0.77
CIP316361.121	Mancozeb = 2	Mancozeb = 3	46.72	0.00	0.00	46.72	70.08	0.00	0.00	70.08	58.40	0.77
CIP316361.187	Mancozeb = 2	Mancozeb = 3	46.72	0.00	0.00	46.72	70.08	0.00	0.00	70.08	58.40	0.77
CIP316361.190	Mancozeb = 2	Mancozeb = 3	46.72	0.00	0.00	46.72	70.08	0.00	0.00	70.08	58.40	0.77
CIP316361.191	Mancozeb = 2	Mancozeb = 3	46.72	0.00	0.00	46.72	70.08	0.00	0.00	70.08	58.40	0.77
CIP316361.244	Mancozeb = 2	Mancozeb = 3	46.72	0.00	0.00	46.72	70.08	0.00	0.00	70.08	58.40	0.77
CIP316367.118	Mancozeb = 2	Mancozeb = 3	46.72	0.00	0.00	46.72	70.08	0.00	0.00	70.08	58.40	0.77
CIP316367.134	Mancozeb = 2	Mancozeb = 3	46.72	0.00	0.00	46.72	70.08	0.00	0.00	70.08	58.40	0.77
CIP316367.148	Mancozeb = 2	Mancozeb = 3	46.72	0.00	0.00	46.72	70.08	0.00	0.00	70.08	58.40	0.77
CIP316375.101	Mancozeb = 2	Mancozeb = 3	46.72	0.00	0.00	46.72	70.08	0.00	0.00	70.08	58.40	0.77
CIP316344.165	Mancozeb = 2	Mancozeb = 4	46.72	0.00	0.00	46.72	93.44	0.00	0.00	93.44	70.08	0.72
CIP316346.204	Mancozeb = 2	Mancozeb = 4	46.72	0.00	0.00	46.72	93.44	0.00	0.00	93.44	70.08	0.72
CIP316352.122	Mancozeb = 3	Mancozeb = 3	70.08	0.00	0.00	70.08	70.08	0.00	0.00	70.08	70.08	0.72
CIP316353.148	Mancozeb = 2	Mancozeb = 4	46.72	0.00	0.00	46.72	93.44	0.00	0.00	93.44	70.08	0.72
CIP316354.112	Mancozeb = 2	Mancozeb = 4	46.72	0.00	0.00	46.72	93.44	0.00	0.00	93.44	70.08	0.72
CIP316355.162	Mancozeb = 2	Mancozeb = 4	46.72	0.00	0.00	46.72	93.44	0.00	0.00	93.44	70.08	0.72
CIP316361.209	Mancozeb = 3	Mancozeb = 3	70.08	0.00	0.00	70.08	70.08	0.00	0.00	70.08	70.08	0.72
CIP316365.166	Mancozeb = 3	Mancozeb = 3	70.08	0.00	0.00	70.08	70.08	0.00	0.00	70.08	70.08	0.72
CIP316367.117	Mancozeb = 2	Mancozeb = 4	46.72	0.00	0.00	46.72	93.44	0.00	0.00	93.44	70.08	0.72
CIP316375.102	Mancozeb = 2	Mancozeb = 4	46.72	0.00	0.00	46.72	93.44	0.00	0.00	93.44	70.08	0.72
CIP316387.156	Mancozeb = 2	Mancozeb = 4	46.72	0.00	0.00	46.72	93.44	0.00	0.00	93.44	70.08	0.72
Kory	Mancozeb = 2	Mancozeb = 2 Cymoxanil = 2 Propineb = 2	46.72	0.00	0.00	46.72	46.72	2.09	47.32	96.13	71.42	0.72
CIP316361.158	Mancozeb = 3	Mancozeb = 4	70.08	0.00	0.00	70.08	93.44	0.00	0.00	93.44	81.76	0.68
CIP316367.177	Mancozeb = 3	Mancozeb = 4	70.08	0.00	0.00	70.08	93.44	0.00	0.00	93.44	81.76	0.68
Amarilis	Mancozeb = 4 Cymoxanil = 4 Propineb = 4	Mancozeb = 3 Cymoxanil = 5 Propineb = 5	46.72	4.18	94.64	145.54	70.08	5.22	118.30	193.60	169.57	0.33
Yungay	Mancozeb = 5 Cymoxanil = 7 Propineb = 7	Mancozeb = 3 Cymoxanil = 6 Propineb = 6	116.80	7.31	165.62	289.73	70.08	6.26	141.96	218.30	254.02	0.00

EIC: Mancozeb = 14.60, Cymoxanil = 8.7, Propineb = 16.9.

Active Ingredient: Mancozeb = 80%, Cymoxanil = 0.06%, Propineb = 70%.

Dose: 2 Kg/ha.

The EIR of late blight resistant clones in Huánuco ranged from 46.72 to 70.08, while it was 289.73 in the susceptible Yungay and 145.54 in the moderately resistant Amarilis. In Oxapampa the breeding clones had an EIR in the range of 46.72 to 93.74, which was lower than that of Yungay and Amarilis with 193.60 and 218.30, respectively (Table 6).

Compared with the EIR of the susceptible variety Yungay, the clones with resistance to late blight reduced their EIR from 68 to 82%, the control variety Kory presented an EIR of 72% and the Amarillis variety 33%.

### Economic profitability

The average economic profitability of the clones resistant to late blight was 189.08% and for Kory, Amarilis and Yungay it was 70.46, 45.45 and 36.50%, respectively. The profitability of the breeding clones was therefore 2.5 to 3 times higher than the controls (Table 7). The lower use of fungicides, less labor and high yields influenced the late blight resistant breeding clones to have a high economic profitability. The sensitivity analysis of costs and yields shows us that the clones must have a minimum yield of 1701.98 kg/ha, while the control cultivars must yield a minimum of 15212.58 kg/ha to prevent loss.

**Table 7:** Economic profitability of potato clones resistant to late blight (2021–2022).

Variety	Late blight resistant breeding clones	Kory (R)	Amarilis (MR)	Yungay (S)
Economic profitability	189.80	70.46	45.45	36.50

R = Resistant to Late blight, MR = Moderately resistant, S= Susceptible.

### Phenotypic stability of marketable tuber yield

**Fig 2:**
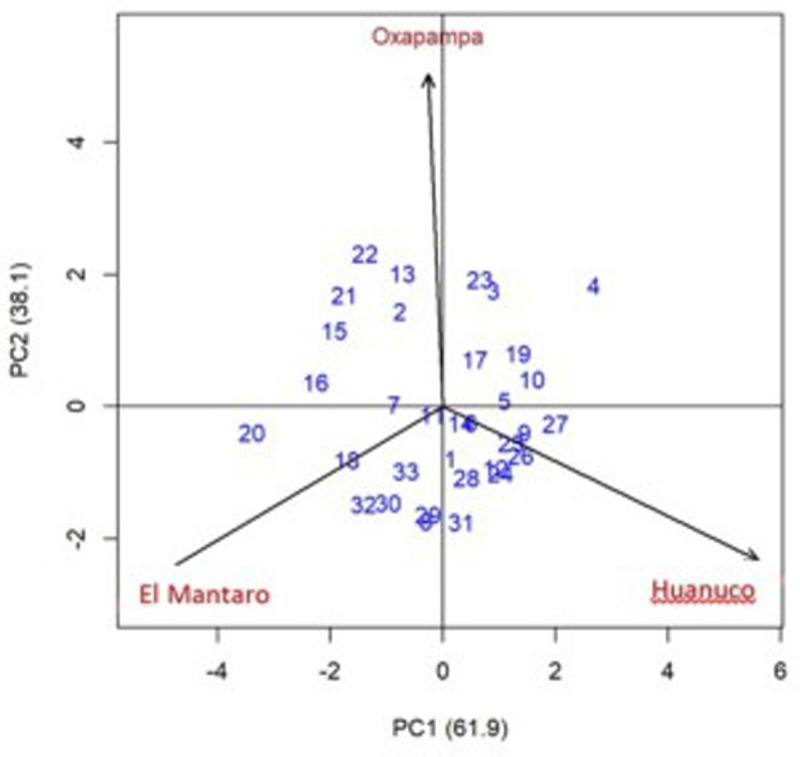
Biplot using principal components 1 and 2 (CP1, CP2) of 30 clones, three control varieties and three locations for marketable tuber yield (2021–2022).

The analysis of variance of the AMMI model for marketable tuber yield (Table 8) shows statistically significant differences (*P* =  0.01) for the sources of variation environments, clones and their interaction (GE), thus indicating that at least one clone has different behavior in the three sites where they were evaluated. This source of variation is very important to determine through the AMMI analysis how many clones have a low GE interaction and are more stable than the others. The principal components PC1 and PC2 were statistically different in the AMMI analysis and explain 61.9% and 38.1%, respectively, of the total GE interaction (Fig 2).

**Table 8:** Additive main effects multiplicative interaction (AMMI) model analysis for marketable tuber yield (2021–2022).

Source of variation	Degrees of freedom	Mean square	Contribution	
			%	Accumulated (%)
Environments	2	4374.6o^**^		
Blocks/Environments	6	171.20		
Clones	32	488.80^**^		
Clones × Environments	64	272.10^**^		
CP1	33	326.50^**^	61.90	61.90
CP2	31	214.10^**^	38.10	100.00
Error	192	70.30		
Total	296			
Coefficient of variation (%)	18.59			

** indicates significant source of variation at *P* ≤  0.01.

The clones are considered stable when the values of the principal components are lower and tend to approach zero. The information obtained through the principal components PC1 and PC2 do not provide a quantitative measure to classify the clones by their phenotypic stability for the yield of marketable tubers. For this reason, the SVAMMI and the MYSI were used to determine which clones are stable and have the highest yields than the susceptible Yungay. This last parameter must have a lower value than that of the Yungay, which had an MYSI of 31 (Table 9).

**Table 9:** Principal components AMMI, Marketable Yield (MY), Stability value AMMI (SVAMMI), Marketable Yield stability Index (MYSI), AUDPC, sAUDPC, Environmental Impact Rate (EIR) in Huánuco, Oxapampa and El Mantaro (Junin) (2021–2022).

#	Clone	PC1	PC2	MY t/ha.	SVAMMI	MYSI	AUDPC	sAUDPC	EIR	Reduction of EIR (%)	Phenotypic Stability
1	CIP316375.102	−0.97	−1.45	63.70	2.14	18	204	0.72	70.08	0.72	Si
2	CIP316361.187	0.57	0.72	56.60	1.17	7	206	0.73	58.40	0.77	Si
3	CIP316367.117	0.66	1.92	54.40	2.20	21	143	0.51	70.08	0.72	Si
4	CIP316356.149	−0.18	−0.13	52.80	0.32	5	262	0.93	58.40	0.77	Si
5	CIP316367.147	1.39	−0.74	52.00	2.37	27	163	0.63	46.72	0.82	Si
6	CIP316367.134	1.21	−0.55	50.80	2.04	23	99	0.52	58.40	0.77	Si
7	CIP316367.177	0.43	−1.07	50.60	1.28	14	372	1.32	81.76	0.68	Si
8	CIP316346.204	0.89	1.76	49.60	2.28	29	111	0.39	70.08	0.72	Si
9	CIP316354.112	−0.30	−1.74	49.00	1.81	23	219	0.78	70.08	0.72	Si
10	CIP316367.132	1.02	−1.01	48.40	1.94	27	178	0.35	58.40	0.77	Si
11	CIP316361.118	0.32	−0.25	47.60	0.57	16	162	0.57	58.40	0.77	Si
12	CIP316353.148	0.51	−0.25	45.30	0.86	20	207	0.73	70.08	0.72	Si
13	CIP316353.741	−0.87	0.03	43.00	1.41	26	149	0.53	58.40	0.77	Si
14	CIP316375.101	−0.26	−1.64	42.80	1.69	29	94	0.33	58.40	0.77	Si
15	Amarilis	−0.14	0.78	40.60	0.81	29	1126	3.99	169.57	0.33	Si
16	Yungay	−0.65	−0.98	41.50	1.44	31	1441	6.00	254.02	0.00	No
17	CIP316361.191	1.33	0.81	48.30	2.30	33	88	0.31	58.40	0.77	No
18	CIP316361.190	−1.71	−0.81	50.10	2.89	35	207	0.74	58.40	0.77	No
19	CIP316360.241	−0.72	2.02	46.40	2.33	36	126	0.45	58.40	0.77	No
20	CIP316361.158	−2.27	0.37	50.90	3.70	37	467	1.66	81.76	0.68	No
21	CIP316358.214	0.93	−0.93	40.10	1.77	38	239	0.85	58.40	0.77	No
22	CIP316355.162	1.58	0.41	45.00	2.60	41	226	0.80	70.08	0.72	No
23	CIP316352.152	1.09	0.09	30.00	1.77	42	189	0.67	58.40	0.77	No
24	CIP316387.156	0.33	−1.75	37.50	1.83	42	318	1.13	70.08	0.72	No
25	CIP316367.148	2.00	−0.26	44.60	3.26	46	161	0.63	58.40	0.77	No
26	CIP316344.165	−0.76	1.44	28.10	1.89	47	225	0.80	70.08	0.72	No
27	Kory	−1.40	−1.48	40.90	2.71	50	353	1.25	71.42	0.72	No
28	CIP316354.169	1.45	−0.39	39.80	2.39	51	213	0.76	58.40	0.77	No
29	CIP316361.244	−1.75	1.69	41.90	3.31	51	64	0.23	58.40	0.77	No
30	CIP316365.166	−1.38	2.32	41.30	3.22	51	420	1.49	70.08	0.72	No
31	CIP316361.209	−3.40	−0.40	42.20	5.53	54	428	1.52	70.08	0.72	No
32	CIP316361.121	−1.93	1.16	37.30	3.34	60	383	1.36	58.40	0.77	No
33	CIP316352.122	2.68	1.84	35.10	4.72	63	520	1.84	70.08	0.72	No

**Fig 3:**
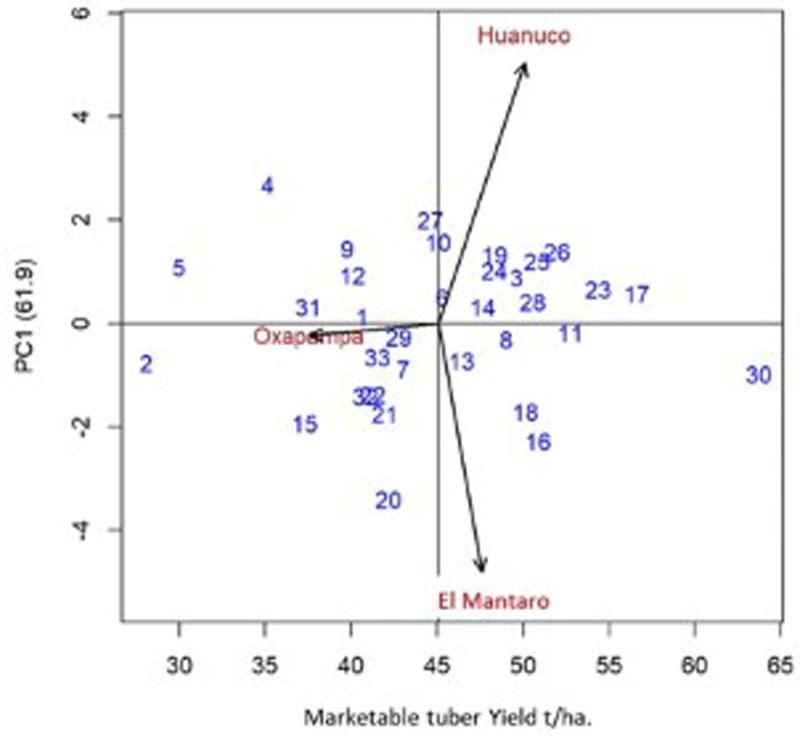
Biplot of potato clones of marketable tuber yield versus principal component 1 (PC1) in three locations in Peru (2021–2022).

Fourteen clones had the lowest MYSI that ranged from 5 to 29 and whose marketable tuber yield under late blight control ranged from 40.6 to 63.7 t/ha. According to the AMMI analysis, these clones had a lower GE interaction or at least lower than the GE interaction of the susceptible Yungay (Table 9, Fig 3). The breeding clone CIP316375.102 had the highest yield of marketable tubers (63.7 t/ha) higher than the yields of Yungay, Kory and Amarilis by 41.5, 40.6 and 40.9 t/ha, respectively. The clones with phenotypic stability are those that have had the most stable marketable tuber yields throughout the three locations. Fig 3 shows the biplot of the marketable yield against the main component 1 (CP1), which contributed with 61.9% of the GE interaction. The breeding clone CIP316361.209 is not phenotypically stable, but is better adapted to El Mantaro (Fig 3).

## Discussion

The AUDPC and sAUDPC were lower in Huánuco probably because of the weather conditions of temperature, relative humidity and precipitation, as well as the probable presence of *P. infestans* isolates different from those in Oxapampa according to [17]. However, these weather conditions were sufficient to induce a high disease pressure in the susceptible variety Yungay. However, it was necessary to control late blight in a timely and adequate manner to achieve economically profitable yields. Twelve applications were carried out during the vegetative period in Huánuco and nine in Oxapampa coinciding with [10], who mentions that in some places with high disease pressure up to 20 applications are needed during the vegetative period of the crop, thus causing a high rate of environmental impact due to the greater use of fungicides compared to clones that received two to four applications depending on their level of resistance as mentioned by [20,21]. In Huánuco, the environmental impact rate of resistant clones to late blight was 6 times less than the EIR of Yungay and three times less than Amarilis, while in Oxapampa it was 4 and 2.5 times with respect to the EIR of Yungay and Amarilis respectively, because of the lower use of fungicides.

The low EIR found in the clones resistant to late blight, due to the lower use of fungicides to control the disease, compared to the susceptible varieties Yungay and Amarilis that had a high IER, due to the greater amount of application of fungicides used for the control of late blight and that allows obtaining economically profitable yields, while the clones with a minimum use of fungicides obtained high tuber yield, even if we compare the yield of the clones in experiment 1, where the clones and varieties only received two applications of Mancozeb and the yield in experiment 2, where the clones received two to four applications of Mancozeb and the varieties up to twelve applications of contact and systemic fungicides, the increase in the yield of the clones was very low compared to the varieties that did have a significant increase in their yields, at the cost of a high EIR, coinciding with [19], who mentions that the high use of agrochemicals puts public health at risk and its impact on the environment,.

The clone CIP396367.147 managed to reduce the EIR by 82% with respect to the EIR of Yungay, while the rest of the clones reduced the EIR between 68 to 77%. This information allows us to verify that it is possible to reduce the environmental impact rate with the use of late blight-resistant potato clones that require a minimum of applications with contact fungicides to achieve economically profitable yields, for the benefit of preserving the environment. The health of producers also benefits by being less exposed to contact with agrochemicals and consumers by having a product with minimal agrochemical residues. In addition, the lower use of fungicides in resistant clones allows to reduce production costs and therefore increase profitability. These clones can be incorporated into sustainable production systems such as family farming since they have positive effects on the environment, increase profitability and improve the quality of life of producers.

The profitability of late blight resistant clones was higher than the control varieties, due to the lower use of fungicides, labor and the higher tuber yield compared to the lower yield in the susceptible control varieties such as Yungay and Amarilis, allowing producers to significantly increase their profitability when using these clones in commercial production.

The stability of tuber yield in clones with resistance to late blight is very important. In the AMMI analysis, clones with MTSI values lower and higher than the susceptible variety Yungay were found, one of the varieties widely planted by farmers and preferred by consumers in Peru. According to [42], clones with low MTSI values are those that are more stable and with high yields, considering this criterion for the selection of clones with values below the MTSI value of the Yungay variety.

## Conclusions

Fourteen clones were selected for based on their high resistance to late blight, low EIR, high economic profitability and phenotypic stability for the marketable tuber yield.

Clones CIP316375.102, CIP316361.187, CIP316367.117, CIP316356.149, CIP316367.147 were the ones that presented the highest tuber yield, phenotypically stable, high resistance to Late blight, low environmental impact and high economic profitability, superior to control cultivars.

These potential new cultivars could contribute to preserving the environment, while also being economically profitable, this would improve the standard of living, particularly for small and medium-sized potato producers in Peru.
