# Supplementary material for: Environmental impact and phenotypic stability in potato clones resistant to late blight Phytophthora infestans (Mont) de Bary, resilient to climate change in Peru
Source: PLoS One. 2025 Feb 11;20(2):e0318255. doi: 10.1371/journal.pone.0318255 (PMC11813132; doi:10.1371/journal.pone.0318255)
Supplement: Attachment — Submitted filename: Review notes KOUAM (Ms. Ref. No.PONE-D-24-45529.docx [file pone.0318255.s001.docx]

Reviewer Comments to Author:

Ms. Ref. No.: PONE-D-24-45529

Ms. Full Title: Environmental impact and phenotypic stability in potato clones resistant to late blight Phytophthora infestans (Mont) de Bary, resilient to climate change in Peru

The authors conducted a study on the Environmental impact and phenotypic stability in potato clones resistant to late blight. Using total tuber yield, marketable tuber yield, evolution of disease intensity over time (AUDPC, sAUDPC), they analyzed a comprehensive 30 potatoes clones for phenotypic stability and economic profitability across locations. Late blight resistance was evaluated in a trial with no chemical application while the application of chemical in controlling late blight helped in evaluation environmental impact. The study was carried out on one season across several location, which is an admirable effort. However, substantial revisions are required if the manuscript is to be considered for publication in this valued journal.

**Abstract:**

1. Line 19. You mentioned that the cultivars Yungay, Amaryllis and Kory were used as controls. Are they controls in terms of resistance to late blight, in terms of yield stability or both? It is important to clarify
2. Line 21. You mention that fourteen clones were resistant, stable phenotypically, low environmental impact and high profitability. Are they having all those characteristics at the same level? Likely not. It is good to mention the main clones. You can mention the top five if you can’t mention all in decreasing levels

**Introduction:**

1. In the last paragraph in the introduction part containing the aims of the study. The authors should change the objectives and project there the different hypothesis that are tested in the study emphatically.
2. The novelty of the study over published literature has to be highlighted in the last paragraph of introduction.

**Materials and methods:**

1. Line 76. You mentioned that the 30 genotypes tested are resistant to late blight. At 850 masl altitude. What was the basis of the selection of those genotypes? Why are you screening against for the resistance? If there is any reason for that, it has to be clearly hypothesized at the introduction
2. Line 79: The controls you used: Amarilis (moderately resistant), Yungay (susceptible) and Kory (resistant). These characteristics where supposed to be the same when using those varieties at your study site?
3. Line 86: precise the precipitation, relative humidity and temperature that give these optimal conditions
4. Line 141-142. You wrote environmental Impact rate (IA) . IA is it the abbreviation?
5. Line 143: All the elements for the EIR formula are not defined. Defined PAI and NA on the formula

**Results:**

1. Line 220, Table 4. Values for AUDPC and sAUDPC are they fixed values or means of values? If they are means of values, provides Standard Deviation so that we can appreciate the significance from the Tukey means separation test. If is fixed values, how do the Tukey test was performed?
2. Line 254: The title of Figure 1 is in Spanish, not in English. You have to harmonize
3. Line 271. Table 5. Why you dis not performed the Tukey Test for the mean separation in order to compare clones as you did with results on AUDPC and sAUDPC provided in Table 4?
4. Table 5: Can we also identify resistant clones from this Table? Are they those with less increase when controlling the disease? If so, provide a relationship between AUDPC values and increase in yield
5. Line 274. Results presented in Figure 2 look appearing in Table 5. Duplication of results. You should choose to presents those results in a Table or in Figure
6. Line 312. Table 6. Could you provide mean separation for EIR for the different genotypes? It will help differentiating the different clones

**Conclusion:**

Provide a conclusion for your research. You should come out with the most promising clones resistant to late blight, with important yield, stable across environments and with higher economic profitability

.
