# Supplementary material for: Environmental impact and phenotypic stability in potato clones resistant to late blight Phytophthora infestans (Mont) de Bary, resilient to climate change in Peru
Source: PLoS One. 2025 Feb 11;20(2):e0318255. doi: 10.1371/journal.pone.0318255 (PMC11813132; doi:10.1371/journal.pone.0318255)
Supplement: Attachment — Submitted filename: RESPONSE TO EDITOR.docx [file pone.0318255.s002.docx]

**RESPONSE TO EDITOR**

**1. We note your Data Availability statement: "All files are available from the**

[**https://na01.safelinks.protection.outlook.com/?url=https%3A%2F%2Fdata.cipotato.org%2Fdataset.xhtml%3FpersistentId%3Ddoi%3A10.21223%2FBW8LWJ&data=05%7C02%7C%7C9c6d10b0cdb64904f5d308dd18d1b59c%7C84df9e7fe9f640afb435aaaaaaaaaaaa%7C1%7C0%7C638694011169339820%7CUnknown%7CTWFpbGZsb3d8eyJFbXB0eU1hcGkiOnRydWUsIlYiOiIwLjAuMDAwMCIsIlAiOiJXaW4zMiIsIkFOIjoiTWFpbCIsIldUIjoyfQ%3D%3D%7C0%7C%7C%7C&sdata=WSmhiw0DRkmWVGg2%2FJ4EYvV%2FYZ0Yx6%2FQTcUOyYZkT2c%3D&reserved=0"**](https://na01.safelinks.protection.outlook.com/?url=https%3A%2F%2Fdata.cipotato.org%2Fdataset.xhtml%3FpersistentId%3Ddoi%3A10.21223%2FBW8LWJ&data=05%7C02%7C%7C9c6d10b0cdb64904f5d308dd18d1b59c%7C84df9e7fe9f640afb435aaaaaaaaaaaa%7C1%7C0%7C638694011169339820%7CUnknown%7CTWFpbGZsb3d8eyJFbXB0eU1hcGkiOnRydWUsIlYiOiIwLjAuMDAwMCIsIlAiOiJXaW4zMiIsIkFOIjoiTWFpbCIsIldUIjoyfQ%3D%3D%7C0%7C%7C%7C&sdata=WSmhiw0DRkmWVGg2%2FJ4EYvV%2FYZ0Yx6%2FQTcUOyYZkT2c%3D&reserved=0%22)

**Unfortunately, this information yields no results. Please confirm that you have provided the correct DOI/link and that your data is set to be publicly available. We hope to hear from you soon with updated information.**

**The link and the DOI are correct, until December 10th it was in a migration process, currently it is operational and you can access the dataset.**
